# Supplementary figures and images for: Ras protein abundance correlates with Ras isoform mutation patterns in cancer
Source: Oncogene. Author manuscript; Available in PMC 2023 Apr 8. (PMC10079525; doi:10.1038/s41388-023-02638-1)

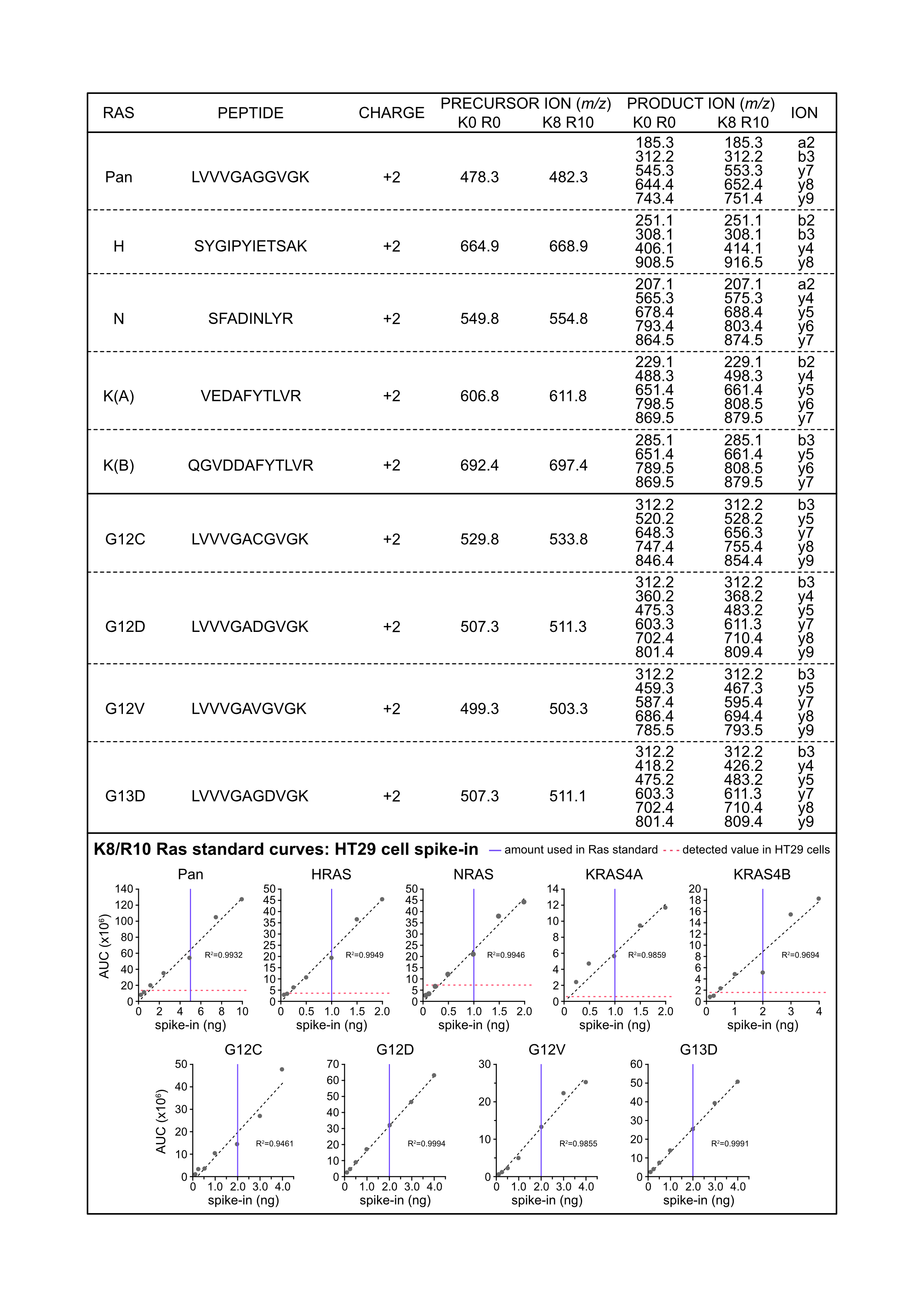

Supplement: Supplementary Figure 1 [file EMS166836-supplement-Supplementary_Figure_1.tif]

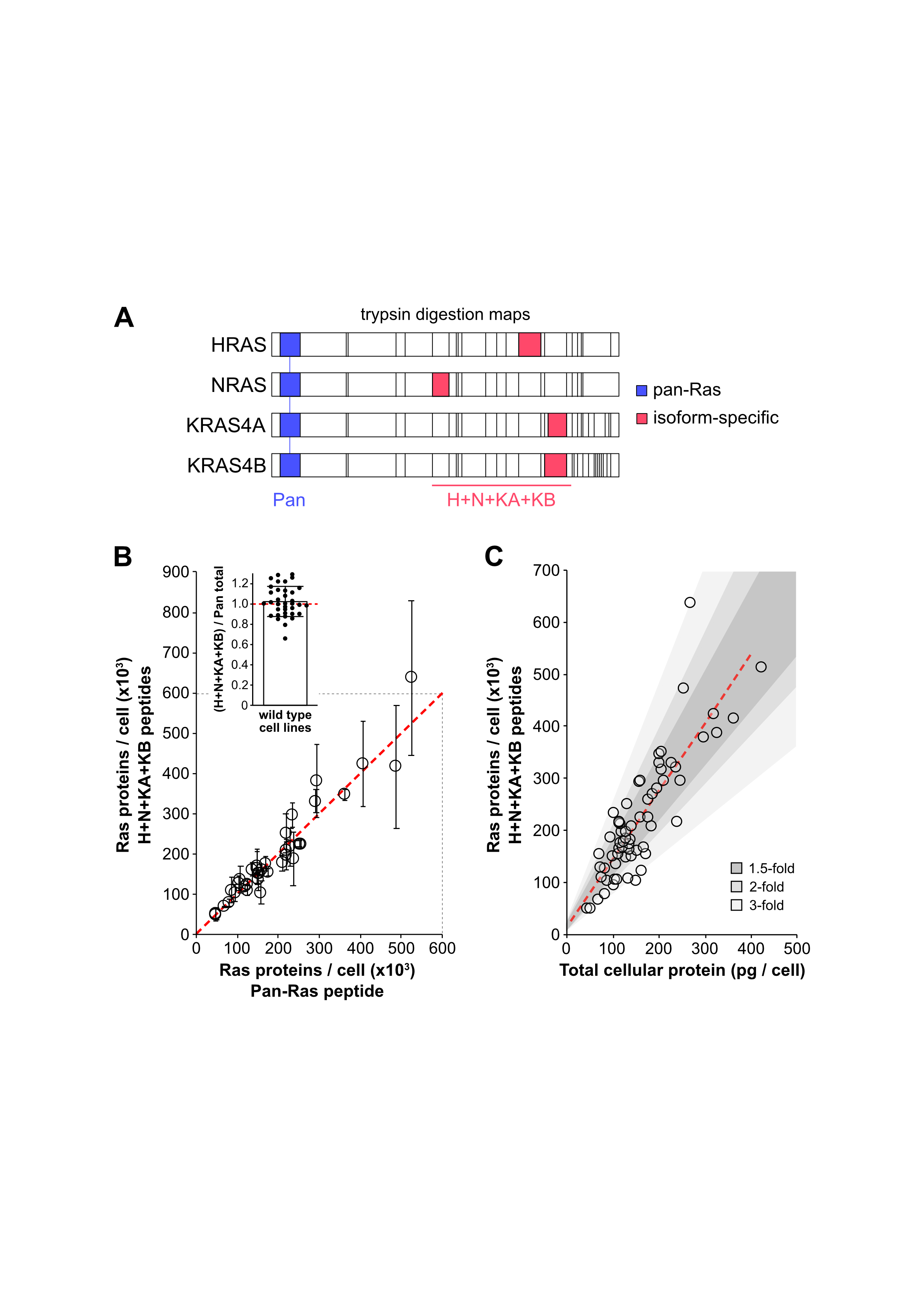

Supplement: Supplementary Figure 2 [file EMS166836-supplement-Supplementary_Figure_2.tif]
