# Supplementary material for: Ras protein abundance correlates with Ras isoform mutation patterns in cancer
Source: Oncogene. Author manuscript; Available in PMC 2023 Apr 8. (PMC10079525; doi:10.1038/s41388-023-02638-1)
Supplement: Supplementary legends [file EMS166836-supplement-Supplementary_legends.docx]

**Ras protein abundance correlates with Ras isoform mutation patterns in cancer.**

**SUPPLEMENTARY MATERIALS**

Fiona E. Hood^1^, Yasmina M. Sahraoui^1^, Rosalind E. Jenkins^2^ and Ian A. Prior^1,3^

Running Title: Quantifying Ras dosage

Keywords: KRAS, PSAQ, rare codon, cancer

Competing interests: The authors have no competing financial or any other conflicts of interests to declare.

^1^ Department of Molecular Physiology and Cell Signalling, Institute of Systems, Molecular and Integrative Biology, University of Liverpool, L69 3BX, UK.

^2^ Centre for Drug Safety Science Bioanalytical Facility, Department of Pharmacology and Therapeutics, Institute of Systems, Molecular and Integrative Biology, University of Liverpool, L69 3BX, UK.

^3^ Corresponding Author: Ian A. Prior

Email: iprior@liv.ac.uk

Tel: +44-151-794-5332

Fax: +44-151-794-4434

**Supplementary Figure 1.** *Transitions for proteotypic Ras peptides and integrated detection sensitivity.* Transitions chosen for endogenous (Lys0 Arg0) and isotope-labelled (Lys8 Arg10) Ras peptides. A linear relationship is observed (R^2^ correlation coefficient > 0.94) for detecting and quantifying Ras isoforms over a wide range corresponding to those observed for endogenous Ras in cell lines.

**Supplementary Figure 2.** *Correlating Ras peptides and total Ras with cell size.* Tryptic digestion maps of Ras isoforms with the locations of pan and isoform-specific peptides indicated (**A**). The pan-Ras peptide measures total Ras in wild type cells. Pan and H+N+KA+KB peptides closely correlate in all wild type cell lines; their ratios average 1.00 as expected if quantification of all peptides is accurate (**B**)**.** Average protein abundance per cell is used as a proxy for cell size. Total Ras abundance and cell size highly correlate (R^2^ correlation coefficient = 0.73) (**C**). Almost all cell values lie within a 3-fold range centered on the trend line. All measurements represent mean ± SEM of n=3 independently processed and analyzed cell samples.

**Supplementary Table 1.** *Ras protein measurements in cells and tissues.* Cell lines, mutation and amplification status and source are all described. Collated means ± SEM of n=3 independently processed and analyzed samples from 78 cell lines and 13 tissues used for all Figures are provided. All source data from biological replicates are included.
